# Community mobilisation with women's groups facilitated by Accredited Social Health Activists (ASHAs) to improve maternal and newborn health in underserved areas of Jharkhand and Orissa: study protocol for a cluster-randomised controlled trial

**DOI:** 10.1186/1745-6215-12-182

**Published:** 2011-07-25

**Authors:** Prasanta Tripathy, Nirmala Nair, Rajendra Mahapatra, Shibanand Rath, Raj Kumar Gope, Suchitra Rath, Aparna Bajpai, Vijay Singh, Vikash Nath, Sarfraz Ali, Alok Kumar Kundu, Dibarkar Choudhury, Sanjib Ghosh, Swati Sarbani, Rajesh Sinha, Christina Pagel, Anthony Costello, Tanja AJ Houweling, Audrey Prost

**Affiliations:** 1 Ekjut, Ward Number 17, Plot 556B, Potka, Po-Chakradharpur, District West Singhbhum, Jharkhand, 833102, India; 2 Clinical Operational Research Unit, Department of Mathematics,4 Taviton Street, London, University College London, WC1H 0BT, UK; 3 UCL Centre for International Health and Development, Institute of Child Health, 30 Guilford Street, London WC1N 1EH, UK

## Abstract

**Background:**

Around a quarter of the world's neonatal and maternal deaths occur in India. Morbidity and mortality are highest in rural areas and among the poorest wealth quintiles. Few interventions to improve maternal and newborn health outcomes with government-mandated community health workers have been rigorously evaluated at scale in this setting.

The study aims to assess the impact of a community mobilisation intervention with women's groups facilitated by ASHAs to improve maternal and newborn health outcomes among rural tribal communities of Jharkhand and Orissa.

**Methods/design:**

The study is a cluster-randomised controlled trial and will be implemented in five districts, three in Jharkhand and two in Orissa. The unit of randomisation is a rural cluster of approximately 5000 population. We identified villages within rural, tribal areas of five districts, approached them for participation in the study and enrolled them into 30 clusters, with approximately 10 ASHAs per cluster. Within each district, 6 clusters were randomly allocated to receive the community intervention or to the control group, resulting in 15 intervention and 15 control clusters. Randomisation was carried out in the presence of local stakeholders who selected the cluster numbers and allocated them to intervention or control using a pre-generated random number sequence. The intervention is a participatory learning and action cycle where ASHAs support community women's groups through a four-phase process in which they identify and prioritise local maternal and newborn health problems, implement strategies to address these and evaluate the result. The cycle is designed to fit with the ASHAs' mandate to mobilise communities for health and to complement their other tasks, including increasing institutional delivery rates and providing home visits to mothers and newborns. The trial's primary endpoint is neonatal mortality during 24 months of intervention. Additional endpoints include home care practices and health care-seeking in the antenatal, delivery and postnatal period. The impact of the intervention will be measured through a prospective surveillance system implemented by the project team, through which mothers will be interviewed around six weeks after delivery. Cost data and qualitative data are collected for cost-effectiveness and process evaluations.

**Study registration:**

ISRCTN: ISRCTN31567106

## Background and rationale

### Millennium development goals 4 and 5

Little time is left to achieve Millennium Development Goals (MDGs) 4 and 5 for maternal and child survival. Only 19 of 68 priority countries are on track to achieve MDG4, which calls for a two-thirds reduction in under-five mortality rates from 1990 levels [1]. Because neonatal deaths account for 41% of under-5 deaths, achieving MDG 4 requires scaling up strategies to reduce neonatal mortality [2]. Effective interventions have long been identified, and key questions now concern the best ways to increase their coverage in an equitable manner, particularly in countries and communities with high mortality rates [3-5].

### Maternal and child survival in India

Around a quarter of the world's neonatal and maternal deaths occur in India [6]. The current neonatal mortality rate (NMR) is estimated at 34.3 per 1000 live births [7] and the maternal mortality ratio at 254 per 100 000 live births [8]. The child mortality rate declined from 33.5 (24.1-45.4) deaths per 1000 live births in 1990 to 13.6 (9.0-19.7) in 2010, and the maternal mortality ratio from 523 (310-835) to 254 (154-395) between 1990 and 2008 [7,8]. To meet MDG4 however, an accelerated reduction in neonatal mortality is required as neonatal deaths account for 55% of under-five deaths [1]. Neonatal mortality rates vary widely between states, ranging from 11 in Kerala to 48 in Uttar Pradesh [9]. They also vary within states and between social groups: the NMR in rural areas is about one and a half times that of urban areas, and rates among the poorest wealth quintile are more than double those among the richest [10]. Socio-economically disadvantaged communities such as indigenous or *adivasi *groups (defined in India's demographic surveys as Scheduled Tribes) have particularly high mortality rates: *adivasi *children have a 25% increased risk of dying before the age of five compared to non-adivasi children [11].

### Current maternal and child health government programmes in India

The Government of India implements three large intersecting programmes to improve women and children's health. The first is the Reproductive and Child Health II (2005-10) programme, which includes the Integrated Management of Newborn and Childhood Illnesses (IMNCI) at community and facility-levels, promotion of skilled care at birth, Essential Newborn Care training for professionals, and health service strengthening. The second is the Integrated Child Development Services (ICDS) programme, which provides a range of nutrition interventions for women and children. The third is India's flagship National Rural Health Mission programme (2005-2012), including the *Janani Suraksha Yojana *(JSY) maternity incentive scheme under which all pregnant women living below the poverty line receive money to deliver in a health facility and receive postnatal check-ups [12]. The NRHM also finances an estimated 820,000 community health volunteers called Accredited Social Health Activists (ASHAs) in priority states. ASHAs are mandated to disseminate health information, counsel women on issues of reproductive and child health and provide essential supplies and drugs (e.g Oral Rehydration Salts and antimalarials). The ASHA programme provides an important platform for scaling up effective community-based interventions for maternal and newborn survival [13].

### Community-based interventions to improve newborn survival

Several community-based interventions have demonstrated substantial effects on neonatal mortality [14,15]. In the home-based newborn care model developed by SEARCH Gadchiroli [16] a trained community health worker identified pregnant women, conducted group health education, carried out two antenatal and 8-12 postnatal home visits, attended deliveries, gave infants a vitamin K injection, identified and managed high risk infants who had signs of sepsis by providing injectable antibiotics in the home and encouraged appropriate referral. This intervention led to a 70% reduction in neonatal mortality over 10 years of incremental implementation, was replicated with seven NGOs (Ankur study) covering around 80 000 population. Elements from home-based newborn care and home visits have been incorporated into ASHAs' training. Following this and other studies, WHO and UNICEF endorsed home visits with components of home-based newborn care as a strategy to improve newborn survival in low-resource settings [17,18]. There is now a need for operational research to examine the feasibility of scaling up this strategy, with equity as a prime concern [19].

Other models emphasise the importance of community mobilisation and participation to improve maternal and newborn health, either with or without a concurrent programme of home-based newborn care. These interventions are premised on the notion that many maternal and newborn deaths can be avoided through better understanding of health problems, changes in antenatal and newborn care practices, and improvements in community perceptions of, and demand for, health services. Several community mobilisation interventions have used a participatory approach, building on the idea that if mothers and other community members take part in decision-making and bring local knowledge, experiences and problems to the fore, they are more likely to own and sustain solutions to improve their communities' health [20]. The Shivgarh trial in Uttar Pradesh successfully tested a combined approach of home visits with social mobilisation through community change agents, leading to a 54% (RR: 0.46, 95%CI: 0.35-0.60) reduction in neonatal mortality over 15 months [21]. The Makwanpur and Ekjut trials showed reductions of 30% (OR: 0.70, 95% CI 0.53-0.94) and 45% (OR: 0.55, 95%CI: 0.46-0.66) in neonatal mortality through community mobilisation with women's groups [22,23], but it remains unclear whether this impact could be maintained if implemented at scale using existing community health workers or volunteers as women's group facilitators.

In India, the NRHM has called for both an expansion of ASHAs' training in home-based newborn care and for "constituting community-based women empowerment groups [...] with the aim to ensure that the female functionaries - ASHAs, Anganwadi workers and Auxiliary Nurse Midwives--become accountable to and work with these groups to help them realise their well-being and rights." [23] Community mobilisation for health is part of the mandate of ASHAs, but specific strategies for community mobilisation led by these workers have yet to be tested [24].

### Justification for this study

Although several intervention models have shown impacts on neonatal survival in India, we do not know whether any of these can bring improvements when delivered through government-linked community health workers such as ASHAs, nor what the barriers and facilitators to scale up might be. This trial aims to test whether a proven participatory community intervention with women's groups can improve maternal and newborn health when implemented with ASHAs, examine its interaction with home visits for maternal and newborn care, and identify the processes through which the intervention can be successfully delivered and scaled-up.

Although the women's group intervention has already been tested in three trials, we decided to adopt a cluster-randomised controlled design to evaluate its impact with ASHAs for two reasons. First, the community mobilisation intervention has not been tested when delivered by community health workers (CHWs); other programmes combining community mobilisation with essential newborn care and delivered by CHWs were not evaluated with baseline data or a control group [25]. Second, several NRHM interventions (including home visits and JSY) are being implemented in the study areas and only an RCT would allow us to robustly quantify the contribution of community mobilisation to any mortality reduction in this context.

In addition, the study is needed to address key implementation research questions and identify strategies for scaling up. Taking community health worker programmes to scale raises questions about adequate levels of remuneration, training, supervision and motivation. Providing recommendations to address these is critical: in a 2007 study, only 3-12% of children born at home in 5 south Asian and sub-Saharan African countries received a visit from a trained health worker within 3 days of birth [26]. Implementation research is necessary in order to understand how best to implement the intervention with ASHAs and whether community mobilisation will supplement or impede their existing activities.

## Design and methods

### Study design

The study is a cluster-randomised controlled trial. Cluster randomisation was preferred over individual randomization as the intervention is a community mobilisation programme delivered at a village-level and with the potential to influence health outcomes both within and between households. An implementation research component is carried out through the trial's process evaluation.

### Aim

To assess the impact and scalability of a community mobilisation intervention with women's groups facilitated by ASHAs to improve maternal and newborn health outcomes among rural tribal communities of Jharkhand and Orissa

### Objectives

1. To test the impact of a community mobilisation intervention with women's groups led by ASHAs on:

(a) Birth outcomes, including neonatal mortality and stillbirths

(b) Care practices and health care-seeking behaviour for mothers and newborns

2. To examine the contribution of community mobilisation with women's groups to maternal and newborn health outcomes in the context of other government programmes

3. To document the context, delivery and costs of the intervention in order to identify lessons for scale-up

### Primary research question

What is the effect of a community mobilisation intervention led by ASHAs on neonatal and perinatal mortality rates?

### Secondary research questions

What is the effect of the intervention on home care practices and health-care seeking behaviour?

What are the factors to consider in scaling up of this intervention, if successful?

### Trial endpoints

The trial's primary endpoint is the neonatal mortality rate over 24 months. Because we expect a short lag period for this intervention to take effect as groups take some time to discuss the causes of maternal and newborn health problems before implementing strategies, we will include data for the last 24 months of the study (1^st ^January 2011 - 31^st ^December 2012) in the final analysis for the primary endpoint, thereby allowing for a four month lag-period. Additional endpoints for the trial include home care practices and health-care seeking behaviour for mothers and newborns and ASHAs' activities related to home visits. The trial endpoints are listed in Table 1.

**Table 1:** Trial outcomes and indicators

Mortality	
Neonatal mortality rate	Number of Neonatal deaths per 1000 livebirths

Stillbirth rate	Number of Stillbirths per 1000 births

Early neonatal mortality rate	Number of early neonatal deaths (0-6 days) per 1000 livebirths

Late neonatal mortality rate	Number of late neonatal deaths (7-28 days) per 1000 livebirths

Maternal mortality ratio	Number of maternal deaths per 100 000 livebirths

Pregnancy-related mortality ratio	Number of pregnancy-related deaths per 100 000 livebirths

Perinatal mortality rate	Number of perinatal deaths per 1000 births

**Care-seeking practices**	

4+ ANC visits	% of births for which mothers received 3+ ANC visits from a skilled provider (doctor, ANM, or other nurse)

Care-seeking for a problem in pregnancy	% of births for which mother had a problem in pregnancy and sought care from a qualified provider

Institutional delivery	% of births that took place at health facility (public or private)

Birth preparedness	% of births for which mothers who made a plan for the delivery

Skilled Birth Attendance	% of home births for which mothers delivered with a skilled birth attendant (doctor, ANM, or other nurse)

Care-seeking for delivery complications	% of births in which complications were identified for which mother sought skilled care

Maternal postnatal visit (qualified provider)	% of births for which mother received at least one postnatal check-up from skilled provider (doctor, ANM or other nurse)

Infant postnatal visits (qualified provider)	% of births for which child received at least one postnatal check-up from skilled provider (doctor, ANM, other nurse or ASHA)

Care-seeking for infant illness	% of births for which mothers sought care from qualified provider if infant had either fever, diarrhoea or cough in first 6 weeks

**Home care practices**	

Clean delivery practices	For home births only: % of births for which attendant washed their hands with soap % of births for which attendant/mother had a sheet % of births for which attendant had new or boiled blade % of births for which attendant boiled thread % of births for which attendant washed their hands % of births for which attendant put nothing/antiseptic on cord

Immediate and exclusive breastfeeding	% of liveborn infants breastfed within first hour % of liveborn infants exclusively breastfed for first six weeks

Thermal care	% of liveborn infants wrapped within first hour after birth % of liveborn infants wiped within first hour after birth % of liveborn infants not bathed in first 24 hours after birth

Skin-to-skin/kangaroo care	% of liveborn infants given kangaroo (skin to skin care) within the first hour after birth

**Interaction with other interventions**	

Home visits & JSY	% mothers who received home visits on the 1^st ^day of life % mothers who received home visits on 1^st^, 3^rd ^and 7^th ^day of life % mothers who had an institutional delivery assisted by the Janani Suraksha Yojana scheme

### Selection of clusters and unit of randomisation

**Figure 1:**
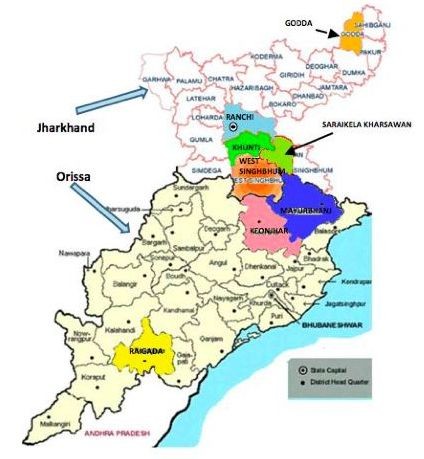
**Study area**.

**Figure 2:**
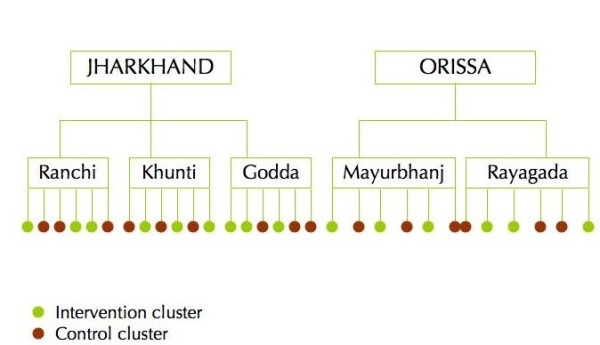
**Trial design**.

The trial area covers a population of approximately 158,053 (based on 2001 Indian Census data) in three districts of Jharkhand (Ranchi, Khunti and Godda) and two districts of Orissa (Mayurbhanj and Rayagada). The location of study areas is shown in Figure 1 and district characteristics in Table 2. The unit of randomisation is a rural geographic cluster of approximately 5000 population. Within each district, the intervention team mapped villages and formed 6 clusters constituted of 5-8 villages and their neighbouring hamlets, with at least 10 active ASHAs per cluster. Within each district, 6 clusters were randomly allocated to receive the community intervention or to the control group, as described in Figure 2. Intervention and control clusters are separated either by natural boundaries (rivers or hills), or by 'buffer villages' not enrolled in the trial.

**Table 2:** Study area characteristics

District	Population*	ASHAs (n)	Villages (n)
Ranchi	31,135	104	35
Godda	29,097	42	41
Rayagada	34,144	56	99
Mayurbhanj	31,913	50	52
Khunti	31,764	66	71

Total	158,053	318	298

* 2009 projection from 2001 Indian Census

### Setting

The study takes place in two districts of Orissa and three districts of Jharkhand, two states of eastern India. The five districts are largely rural and have a high proportion of *adivasi *people. The main tribal groups in the trial area are the Ho, Munda, Oraon, Santhal, Binjhiya, Paharia, Bhumij, Bhatudi, Bhuyian, Gond, Mankidia and Kondh. In previously published research carried out in other rural tribal areas of these states, [22] we found a neonatal mortality rate of 58 per 1000 livebirths and a maternal mortality ratio of 510 per 100 000 livebirths. Around 35% of mothers had three or more antenatal check-ups and only 17% delivered in a health facility. In December 2010, the recruitment of ASHAs had been completed in Jharkhand and Orissa but their training in home-based newborn care had not begun. JSY was operating in both states but had higher coverage in Orissa [13]. The trial is being implemented by Ekjut, an NGO that has been working in Jharkhand and Orissa since 2003, in collaboration with the Centre for International Health and Development at University College London (UK).

### Target group and eligibility criteria

The target population will be rural, mainly tribal communities with poor access to health services. The target group for the women's group intervention is women of childbearing age between the ages of 15 and 49 years, and particularly pregnant mothers. However the groups are open to all community members, including adolescent girls, older women and men. Community health workers may also participate in group discussions. Participants in the trial will be all women who have given birth in the study area between 1^st ^September 2009 and 31^st ^of August 2010 for the baseline period, and between 01^st ^of September 2010 and 31^st ^December 2012 for the trial period, and who have agreed to take part in the study when approached by an interviewer around six weeks after delivery. Excluded are women who decline to be interviewed or have migrated out of the study clusters, and, for secondary outcomes (care-seeking and home care practices), women who gave birth in the study area but who cannot be traced after 9 months. During an earlier trial, 1.3% of births detected by a similar surveillance system to the one used in this study (255 out of 19068) were to mothers who migrated in or out of the study clusters, and we were not able to obtain background socio-economic data on these migrants. The intention to treat population (for the primary outcome) will therefore be all mothers who reside in the study clusters and have given birth in the study area in the last 24 months of the intervention and did not migrate out of the study clusters. Participation in intervention activities will be voluntary and women are free to join or leave a group at any time.

### Randomisation and allocation

The randomisation was carried out as follows: in each district, we invited stakeholders from each cluster, including Village Health Committee members and staff from other NGOs to a meeting. We allocated a number to each cluster, wrote these numbers on small plastic balls and placed the balls within a dark bag. The intervention team reminded stakeholders about the aim and length of the intervention and about the purpose of the randomization process. We asked each of the participants to draw one ball from the bag and read the cluster number out until all balls had been picked. The numbers were then written on a sheet of paper in the order of selection. Finally we placed twenty pieces of paper numbered 1 to 20 - each corresponding to a unique allocation sequence generated by CP in Excel - in the dark bag and asked a participant to select a paper and read out the number. The corresponding sequence was then used to publicly allocate each cluster to one of two groups to ensure transparency among the stakeholders and also to avoid conflict as only intervention area ASHAs would be entitled to incentives.

#### The interventions

##### Women's groups

The intervention is a participatory learning and action cycle of 20 meetings, during which women's groups open to other community members identify and prioritise maternal and newborn health problems in their area, identify strategies to address these, implement these strategies and evaluate the entire process. This cycle was adapted from an earlier intervention tested during the Ekjut trial and in other settings [27,28]. Specific adaptations based on lessons from the Ekjut trial included a minimum recruitment of pregnant women into groups and an emphasis on care for newborns during winter, as a strong increase in neonatal deaths was observed in the study areas during this season. The team also prioritized materials to be used (e.g. picture cards) by selecting the most important maternal and newborn problems with ASHAs. The cycle consisted of fortnightly meetings for the first four months (Phase 1 and Phase 2), following by monthly meetings after this, as described in Table 3. In the first phase, consisting of three meetings, the ASHA will introduce the project, then help the group identify and prioritise local maternal and newborn health problems. In the second phase, consisting of four meetings, groups discuss causes and solutions to prioritized problems through participatory games and using picture cards, then identify and prioritise strategies for addressing the problems. At the end of this phase the groups prepare to hold a community meeting in which they share their thoughts, problems and strategies with other community members, including men, traditional birth attendants, mothers-in-law and Community Health Workers. In phase three, the groups implement their strategies and carry out practical role-plays focusing on care-seeking and emergencies. Finally, in phase four the groups evaluate each of the phases and progress on their prioritized strategies. The intervention is expected to run for 24 months (20 meetings, two community meetings and two additional months to make up for any cancellations of meetings).

**Table 3:** Intervention meeting plan

ASHA TRAINING 1		
**Phase I: **Problems	Meeting 1	Introduction to the project
	Meeting 2	Identifying & prioritizing maternal problems in the community
	Meeting 3	Identifying & prioritizing neonatal problems in the community

**ASHA TRAINING 2**		

**Phase II:** Strategies	Meeting 4	Thermal care for newborns (story on preventing winter deaths)
	Meeting 5	Understanding causes and solutions for prioritized problems (story focusing on causes, effects and management)
	Meeting 6	Identifying and prioritising strategies for implementation
	Meeting 7	Choosing a method & preparing for sharing at the community meeting
	Meeting 8	Preparing for a community meeting

**COMMUNITY MEETING 1 AND ASHA TRAINING 3**		

**Phase III:** Implementation	Meeting 9	Assigning responsibilities for the implementation of strategies
	Meeting 10	Birth preparedness - Hygienic practices - Essential Newborn Care - Management of twins and low birth weight babies (demonstration)
	Meeting 11	Understanding & implementing home care strategies using picture cards
	Meeting 12	Understanding & implementing preventive strategies using picture cards
	Meeting 13	Understanding & implementing strategies for newborn emergency problems using picture cards
	**ASHA TRAINING 4**	
	Meeting 14	Accessing appropriate care (game)
	Meeting 15	Emergency preparedness for maternal and neonatal problems (role-play)
	Meeting 16	Preventing maternal deaths through campaigning against the first delay
	Meeting 17	Learning about strategies implemented by other groups
	Meeting 18	Preparation for community meeting
		**COMMUNITY MEETING 2**
	**ASHA TRAINING 5**	

**Phase IV:**	Meeting 19	Phase-wise evaluation
Evaluation	Meeting 20	Evaluation of women's group activities

##### ASHA selection, training and incentivisation

In each district we approached district-level ASHA coordinators and Village Health Committees (VHCs), explained the women's group intervention and asked VHCs to identify suitable ASHAs to facilitate the groups. These ASHAs had to be functional according to government criteria: they needed to have undergone at least three training sessions and be village-based. We identified problems that could arise whilst working with ASHAs during the course of the intervention and agreed on strategies to address these. In order to assist the ASHAs, we appointed a co-facilitator who was given an incentive of Rs 50 per meeting to help with record keeping. This co-facilitator was not given training to conduct meetings, and ASHAs were left in charge of facilitation. Should an ASHA not be accepted by her village, we agreed that the district-based intervention team would seek to help her, and, if unsuccessful, ask the co-facilitator to conduct meetings after training. In the event that an ASHA should drop out of the intervention permanently the VHC and women's group would decide whether a new ASHA should be trained, or whether a co-facilitator should take over and be paid the full incentive. The intervention team designed four training sessions for selected ASHAs, one for each phase of the intervention. One cluster coordinator employed by Ekjut is posted in each district. ASHAs are given an incentive of Rs100 per day as in government trainings (US$2) and Rs200 per meeting. A total of 149 ASHAs were selected to implement the intervention.

##### Additional interventions

Community involvement in monitoring health services is an essential component of the NRHM programme. Village health and sanitation committees (VHSCs or VHCs) are mandated to monitor local services as well as disseminate information about rights and entitlements to healthcare. We will undertake three main activities related to strengthening health services in *both *intervention and control areas. First, we will carry out at least one village health committee meeting about rights and entitlements in each village during the study period. Second, we will organise meetings with government officials and hospital management committees to inform the provision of appropriate care for mothers and newborns in facilities in the study districts. Finally, we will carry out at least one meeting with ASHAs using the appreciative inquiry method [29] to strengthen their job motivation and help them further enhance their work performance.

#### Impact evaluation

##### Sample size

A sample size calculation was carried out in June 2010 using 11 months of available data on birth outcomes from the study areas prior to the creation of clusters. Baseline mortality data collected from the study areas between 1^st ^February 2009 and 31^st ^December 2009 indicated an NMR of 66 per 1000. We estimated *k *(the between cluster correlation coefficient) at 0.1 on the basis of data from the Ekjut trial as well as baseline data from the JOHAR trial areas. On the basis of surveillance from existing clusters, for an average cluster population of 5000, we expect an estimated 100 livebirths per cluster per year. We aim to assess the impact of the intervention using a total of 2 years of birth outcomes. We used formulae from Hayes and Bennett to estimate the power of the study [30]. Assuming a more conservative baseline mortality rate estimate of 55 per 1000, with 100 livebirths per cluster per year in 30 clusters, the study will have between 76.6% and 79.6% power to detect a 30% reduction in the NMR (from 55 per 1000 to 38.5 per 1000) over 24 months.

##### Surveillance

**Figure 3:**
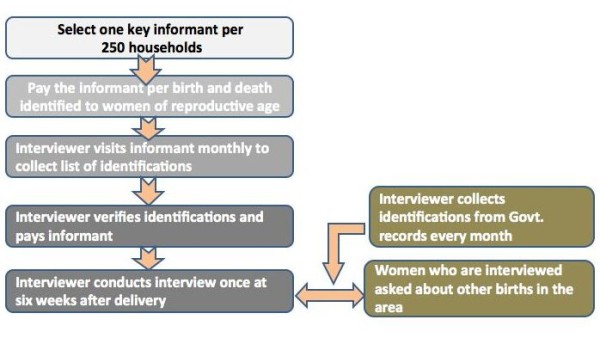
**Trial vital events surveillance system**.

A monitoring team independent from the intervention team was established to collect information on births and deaths to women of reproductive age and on events during the antenatal, delivery and postnatal periods through a surveillance system adapted from a previous study, covering a total population of around 158,053 [31] and described in Figure 3. One key informant (a community member) reports all births and deaths to women of reproductive age to an interviewer. The informant is visited on a monthly basis by an interviewer from the surveillance team. The interviewer checks the report, pays the informant an incentive of Rs30 for an identification and another Rs 50 for conducting an interview with the identified mother around six weeks after the delivery. The collected questionnaires are examined by District Monitoring Coordinators with guidance from District Managers. Completed questionnaires are then entered into a database designed in Microsoft Access. Baseline data on birth outcomes were collected for a full year from 1^st ^September 2009 to 31^st ^August 2010.

##### Data monitoring, masking and analysis plan

Due to the nature of the intervention, the intervention team or participants cannot be blinded to the allocation however the analysts will be blinded to the allocation for the data safety and monitoring board and until the definitive analysis is performed. The intervention and monitoring team hold their meetings on separate days, and the content of the intervention is not discussed with monitoring team members. We shall analyse data for the primary and secondary endpoints for a data safety and monitoring board (DSMB) to be held in late 2011. The DSMB will be undertaken according to the principles stated in the DAMOCLES statement [32]. We will carry out intention-to-treat analyses using individual level data, adjusting for clustering using either logistic regression with random effects or generalised estimating equations [33]. In this data safety and monitoring board we will analyse data for the period from 1^st ^September 2010 till 31^st ^June 2011 (10 months of data). The DSMB will have three objectives:

(1) To report on the observed number of livebirths and neonatal mortality rate in the trial area in order to examine whether the original sample size calculation is robust; if the expected number of livebirths has not been reached the DSMB may recommend extending the trial;

(2) To assess the comparability of trial arms by examining data on the socio-economic characteristics of participants and clusters at baseline, including the coverage of home visits, in order to define any adjustments to be made in the final analysis;

(3) To examine available data for the primary and secondary endpoints in order to decide whether to continue or stop the trial

##### Stopping rules

Because previous trials of women's groups in Nepal and eastern India have shown a strong effect of groups on neonatal mortality, we aim to stop the trial and introduce groups into the control area after the DSMB if we observe a reduction in the NMR of 40% or more after adjusting for differences observed at baseline, or if we observe a reduction inferior to 40% but with improvements in secondary indicators (home care or care seeking practices) consistent with a reduction, and satisfactory implementation quality indicators (70% or more of ASHAs have conducted all meetings at the time of the DSMB; % of women who gave birth in the study area who received information from a group member; % of women of reproductive age in group meetings).

##### Sub-analyses

We will compare the proportion of women who received postnatal home visits by an ASHA and the proportion of women who delivered in institutions among areas with and without ASHA-led mobilisation.

#### Process evaluation

The process evaluation has five objectives:

1. To describe the context in which the intervention was delivered (including the backdrop of NRHM interventions in intervention and control)

2. To describe the intervention in theory (model) and in practice (the way in which it was delivered)

3. To identify factors that facilitate or prevent the delivery of this intervention by ASHAs, and its implementation at scale

4. To develop hypotheses about the mechanisms through which the intervention may have worked, including its interaction with NRHM interventions

5. To compare the mechanisms linked to the success of the NGO facilitator-led intervention with those of the ASHA-led intervention

##### Methods

We will use a mix of quantitative and qualitative methods to address each of these objectives, as described in Table 4. The process evaluation will aim to identify factors that would facilitate scale up.

**Table 4:** Process evaluation objectives, methods and data sources

OBJECTIVE	INDICATORS & ISSUES	METHODS	DATA SOURCE
**1. To describe the context in which the intervention was delivered**			
	Information on terrain, infrastructure and health service provision	Discussions with facilitators, NGO and government staff Quantitative analysis of formats	Context notes and formats M&E data* Meeting reporting formats collated by co-facilitators HSS mapping format Process evaluation manager notes
	Profile of intervention and control communities, cultural practices including health practices, livelihoods including seasonal occupation, and migration	Quantitative analysis Qualitative analysis of discussions with ASHAs and observation notes of project staff	M&E data (including baseline data) Context format Process evaluation manager notes
	Profile of clusters (Population composition, number of ASHAs, population size and spread of ASHA catchment area, number of villages, hamlets and total population)	Quantitative analysis	M&E data Population Census, 2001 Context format
**Objective 2: to describe the intervention in theory and in practice**			
	Intervention plan		Trial protocol
	Profile of ASHAs category, n of training modules completed) Profile of co-facilitators Profile of cluster coordinators	Quantitative analysis	ASHA profile register Cluster coordinator reports ASHA profile register
	ASHA and cluster coordinator recruitment process and training	Qualitative review of recruitment process of cluster coordinator and co-facilitators Qualitative summary of training reports	Interview schedules and process evaluation manager notes District-level training reports (6 × 5), training schedule, materials and games
	ASHAs', cluster coordinators' and co-facilitators' perception of the intervention	Qualitative analysis	Discussion with ASHAs and cluster coordinators at the end of each intervention phase Discussions with co-facilitators by cluster coordinators at the end of the cycle
	Meeting site	Quantitative compilation	Discussion with ASHAs and cluster coordinators at the end of each intervention phase
	Group characteristics	Quantitative analysis	Group description format held by cluster coordinators (name, location, n of meetings, month/year of formation)
	Meeting duration, attendance, member characteristics & group discontinuation	Quantitative analysis	Meeting reporting format Process evaluation manager notes of review meetings with cluster coordinators (for HR issues with ASHAs and co-facilitators) District-level HR register for cluster coordinators, ASHAs & co-facilitators M&E data
	Identification and prioritization of problems	Quantitative analysis	Prioritised problem format (meeting specific) Women's group observation notes (5 groups followed from start to end of cycle)
	Identification and prioritization of strategies	Quantitative analysis	Strategies format (meeting specific) M&E data
	Village-level and cluster-level community meetings (ASHAs' and cluster coordinators' perception)	Qualitative summary	Discussion with ASHAs and cluster coordinators at the end of each intervention phase (1-4) Community meeting format (use for 1^st ^and 2^nd ^community meetings)
	Support given to members for community meetings Methods for obtaining and using resources for the community meetings	Qualitative summary	Review meeting notes
	Members' phase-wise evaluation of intervention	Quantitative analysis	Phase-wise evaluation chart
	Group members' perception of the intervention and its impact	Qualitative analysis of 10 focus group discussions with group members (2 per district)	FGD transcripts
**Objective 3: To identify factors that facilitate or prevent the delivery of this intervention by ASHAs and its implementation at scale**			
	Enablers and barriers to ASHAs delivering the intervention	Qualitative analysis of 5 FGDs (1 per district) at the end of the PLA cycle and women's group case studies Qualitative summary	FGD transcripts and case study notes Reports of ASHAs, coordinators and cluster coordinator
	HR lessons for scaling-up	Qualitative summary	Costing formats (Rajesh Sinha) Notes from review meetings with ASHAs and coordinators
**Objective 4: To develop hypotheses about the mechanisms through which the intervention may have worked**			
		Group discussion with Ekjut team members to review the process evaluation findings and generate a list of hypotheses about the intervention mechanisms	FGD transcripts Quantitative analyses Case studies notes Observation notes Facilitators' registers
**Objective 5: To compare the mechanisms linked to the success of the facilitator-led intervention with those of the ASHA-led intervention**			
	Perception of group members regarding behaviour change among themselves and non-group members	5 group discussions with group members	FGD transcripts Case studies
	Population coverage of groups & coverage of pregnant women as compared with Ekjut trial intervention		Census data Register M&E
	Coordinators' and cluster coordinators' perceptions of intervention mechanisms	Discussion incorporating results of FGDs with group members to obtain coordinators' feedback	Discussion notes
	Workload analysis of facilitators in ASHAs in JOHAR trial	Qualitative summary	Documents from review meetings with ASHAs

* M&E refers to the main trial monitoring and evaluation questionnaire

#### Data management

Quantitative data will be entered in the main office in a relational database on Microsoft Access. All infants born in the study clusters will be given a unique ID. After checking and entry the questionnaires will be stored in a locked room for future reference. Qualitative data will be collected either in note form or audio-recorded. Audio-recordings will be kept on a dedicated, password protected computer by the process evaluation manager. Qualitative focus group discussions and interviews will be transcribed and analysed using a thematic approach.

#### Quality control

One key informant per 250 population will identify births and to women of reproductive age. Each event will be checked by an interviewer, who will pay the key informant an incentive. Surveillance monitors will meet regularly with interviewers both in the field and at the district offices. Accuracy of the quantitative data will be checked first by the interviewer, then by the Monitoring coordinators and District Managers and finally at the time of data entry in the main office. Data cleaning will be carried out internally through systematic checks on key fields (mother ID, baby ID, birth outcome).

#### Strategies to reduce contamination

Contamination may occur if ASHAs or women discuss issues related to maternal and newborn health with each other between clusters. This may happen if ASHAs travel from one cluster to another (for example for training or to meet relatives), or if participants migrate permanently or temporarily to a neighbouring cluster. This may result in information or strategies being shared, the effect of the intervention spreading to control clusters and dilution of differences between treatment arms. In order to reduce the risk of contamination, intervention and control clusters are separated either by natural boundaries (e.g. hills or rivers) or by 'buffer villages'. In the five rural districts where the study will take place, villages are quite distant from each other. We will also ask about exposure to women's groups in control areas so will be able to quantify any contamination.

#### Economic evaluation

All intervention related costs, including start-up and running costs will be audited through the project accounting system. We shall carry out a cost-effectiveness analysis to determine the scalability of the intervention and their replicability within existing government systems. We will also estimate the cost per neonatal death averted.

#### Ethical issues

##### Community consent

We shall seek permission from local community representatives (headmen in Jharkhand and *Panchayati Raj *institution leaders in Orissa) to work with women's groups and the ASHAs, and to collect data in their areas. These representatives were invited to the public randomisation.

##### Individual consent

Individual consent will be sought from each mother approached as a result of a birth or death identification, and consent recorded through a signature or thumbprint.

##### Benefits to the control communities

Because the women's group intervention has demonstrated an impact on neonatal mortality in a similar setting, we have put in place stopping rules in order to introduce the intervention to control areas if positive results are observed at the first data safety and monitoring board. In addition, activities such as strengthening of Village Health Committees and Appreciative Inquiry workshops with ASHAs will be conducted in both intervention and control clusters.

##### Treatment of illness in participating communities

The study team will encourage referral to an appropriate health facility if they identify minor, acute or chronic illness in mothers or infants in either intervention or control areas.

##### Confidentiality of information

All information will be confidential. Access to information will be limited to interviewers and monitors in the field and main office and then to data entry management staff. No publications, analyses or reports will include the names of participants.

##### Sustainability and scalability

One key objective of the study is to identify mechanisms for scaling up the intervention. We will share implementation plans and findings with NRHM implementers in Jharkhand and Orissa. The intervention will be introduced immediately into the control areas if a positive impact is observed at the first Data Safety and Monitoring board. We shall invite a representative of the Indian Council of Medical Research to join the data safety monitoring group.

##### Approval

Ethical approval for the study is being sought through an independent ethical research committee chaired by Dr AK Debdas in Jamshedpur, India. The study was approved by the Research Ethics Committee of University College London (UK) with project identification number 1488/001.

## List of abbreviations

ASHA: Accredited Social Health Activist; CHWs: Community Health Workers; DSMB: Data Safety and Monitoring Board; ICDS: Integrated Child Development Services; IMNCI: Integrated Management of Newborn and Childhood Illnesses; JOHAR: Jharkhand Orissa Health Action Research for maternal and newborn health; JSY: *Janani Suraksha Yojana *(India's Safe Motherhood incentive scheme); MDG: Millennium Development Goal; NGOs: Non-governmental Organisations; NMR: Neonatal Mortality Rate; NRHM: National Rural Health Mission; SEARCH: Society for Education, Action and Research in Community Health (Gadchiroli); UNICEF: United Nations' Children's Fund; VHSCs: Village Health and Sanitation Committees (also Village Health Committees); WHO: World Health Organisation

## Competing interests

The authors declare that they have no competing interests.

## Authors' contributions

NN and PT are the project directors, contributed to the design of the study, lead the management of the trial, and will participate in the analysis and interpretation of data. NN, RM and SR contributed to the design of the study and will coordinate the data collection. RG contributed to the design of the intervention with Village Health Committees and is responsible for its implementation. AP contributed to the study design, will lead the analysis and wrote the first draft of the study protocol. CP, TH and AC contributed to the design of the study and will participate in the analysis and interpretation. AB, VB, ST, SR, DC, VB, SG, SA and SN contributed to the design of the intervention and randomisation, will be responsible for the implementation and will participate in the interpretation of data. AC, PT and NN obtained grant funding. All authors contributed to the critique of the manuscript.
